# High throughput human genotyping for variants associated with malarial disease outcomes using custom targeted amplicon sequencing

**DOI:** 10.1038/s41598-023-39233-z

**Published:** 2023-07-26

**Authors:** Ashley Osborne, Jody E. Phelan, Leen N. Vanheer, Alphaxard Manjurano, Jesse Gitaka, Christopher J. Drakeley, Akira Kaneko, Kiyoshi Kita, Susana Campino, Taane G. Clark

**Affiliations:** 1 Faculty of Infectious and Tropical Diseases, London School of Hygiene & Tropical Medicine, London, UK; 2 School of Tropical Medicine and Global Health, Nagasaki University, Nagasaki, Japan; 3 Mwanza Medical Research Centre, National Institute for Medical Research, Mwanza, Tanzania; 4 Joint Malaria Program, Kilimanjaro Christian Medical Centre, Moshi, Tanzania; 5 Directorate of Research and Innovation, Mount Kenya University, Thika, Kenya; 6 Centre for Malaria Elimination, Mount Kenya University, Thika, Kenya; 7 Department of Parasitology, Graduate School of Medicine, Osaka Metropolitan University, Osaka, Japan; 8 Department of Microbiology, Tumor and Cell Biology, Karolinska Institutet, Stockholm, Sweden; 9 Faculty of Epidemiology and Population Health, London School of Hygiene & Tropical Medicine, London, UK; 10 Department of Infection Biology, London School of Hygiene and Tropical Medicine, Keppel Street, London, WC1E 7HT UK

**Keywords:** Genetics, Disease genetics, Bioinformatics

## Abstract

Malaria has exhibited the strongest known selective pressure on the human genome in recent history and is the evolutionary driving force behind genetic conditions, such as sickle-cell disease, glucose-6-phosphatase deficiency, and some other erythrocyte defects. Genomic studies (e.g., The 1000 Genomes project) have provided an invaluable baseline for human genetics, but with an estimated two thousand ethno-linguistic groups thought to exist across the African continent, our understanding of the genetic differences between indigenous populations and their implications on disease is still limited. Low-cost sequencing-based approaches make it possible to target specific molecular markers and genes of interest, leading to potential insights into genetic diversity. Here we demonstrate the versatility of custom dual-indexing technology and Illumina next generation sequencing to generate a genetic profile of human polymorphisms associated with malaria pathology. For 100 individuals diagnosed with severe malaria in Northeast Tanzania, variants were successfully characterised on the *haemoglobin subunit beta (HBB), glucose-6-phosphate dehydrogenase (G6PD), atypical chemokine receptor 1 (ACKR1)* genes, and the intergenic Dantu genetic blood variant, then validated using pre-existing genotyping data. High sequencing coverage was observed across all amplicon targets in *HBB, G6PD, ACKR1,* and the Dantu blood group, with variants identified at frequencies previously observed within this region of Tanzania. Sequencing data exhibited high concordance rates to pre-existing genotyping data (> 99.5%). Our work demonstrates the potential utility of amplicon sequencing for applications in human genetics, including to personalise medicine and understand the genetic diversity of loci linked to important host phenotypes, such as malaria susceptibility.

## Introduction

Despite decades of progress, malaria incidence in 2020 increased for the first time since the start of the millennium and resulted in an estimated 241 million cases and 627,000 deaths, a 12% increase from the number of deaths reported in 2019^1^. This increased incidence of malaria was largely due to disruptions in implementing malaria and vector control programmes, as well as supply chain failures, caused by the COVID-19 pandemic^1,2^. In addition to the COVID-19 pandemic, increased disease incidence has also linked to the continued emergence and spread of *Plasmodium* parasite drug resistance and mosquito insecticide resistance around the world, and the ongoing influence of climate change on vector populations and weather patterns^3,4^. As malaria continues to be a major burden on public health in low- and middle-income countries, where it disproportionately affects pregnant women and children under 5 years of age, the need for technological advancements in disease control and elimination have never been more apparent^1,5^.

Malaria has exhibited the strongest known selective pressure on the human genome observed in recent history and has been proven to be the driving force behind a variety of human polymorphisms associated with malarial disease outcomes and severity, such as the sickle-cell trait, thalassaemia, glucose-6-phosphatase deficiency (G6PD), and other erythrocyte variations^6,7^. Regarded as the classic paradigm of balancing selection in human populations, the sickle-cell allele (HbS) has evolved independently in multiple malaria-endemic regions due to its ability to confer up to a ten-fold reduced risk of severe malaria*,* despite its pathogenic effects in homozygous carriers^8^. While the sickle-cell allele has been associated with protection against severe disease caused by *Plasmodium falciparum*, the Duffy negative (Fy−) phenotype has all but eliminated *P. vivax* from much of sub-Saharan Africa and is not associated with any pathogenic phenotypes^9^.

Due to the high prevalence of G6PD in sub-Saharan Africa, which results in diminished activity of the G6PD enzyme, it is believed that G6PD genetic variants on the X chromosome arose due to selection pressure exhibited by malaria on the human genome^10,11^. This hypothesis has been corroborated by studies that have identified negative associations with severe malaria in hemizygous males and heterozygous females^7,10^. A majority of individuals carrying this genetic disorder remain asymptomatic, however clinical manifestations of the deficiency can include haemolytic anaemia which can be exacerbated by treatment with primaquine, an antimalarial drug used for the treatment of *P. falciparum* gametocytes and relapses of *P. ovale*^11,12^.

Although progress has been made towards understanding the impacts that variations in the host genome have on malarial disease outcomes, Africa, as a whole, remains underrepresented in genetic studies^13^. Genome-wide studies (e.g., The 1000 Genomes project) have provided invaluable baseline data for human genetics and represented individuals across Indigenous African populations^14^. However, with approximately 2000 ethno-linguistic groups thought to exist across the African continent, more information is needed to gain a comprehensive understanding of the genetic differences between populations and their implications on disease^13^. Additionally, this underrepresentation of individuals with African ancestry in genetic databases poses challenges towards the successful application of genetically tailored medicine as it becomes more widely available with rapid advances being seen in sequencing technology^13,15^.

Genome-wide association studies (GWAS) test for differing genotype or allele frequencies of millions of genetic variants between phenotypes, accounting for the confounding effects of population structure^16^. Variants known to confer phenotypes associated with severe malarial disease, such as HbS and G6PD deficiency, can be measured and their impact on the human genome quantified. This includes investigation into allelic heterogeneity and the implications of differing phenotypes on disease. An example of this being the complexity surrounding G6PD deficiency, with heterozygous females and hemizygous males exhibiting protection from severe malaria, while homozygous individuals range from having no added protection to being more at-risk for severe disease^7^. Perhaps one of most beneficial aspects of GWAS studies is the ability to screen for novel loci with associations to disease outcome, such as in case–control studies, leading to the identification of new “candidate genes” for malaria sensitivity to be investigated further^17,18^.

Large-scale, multi-population, GWAS studies have highlighted the high degree of genetic diversity at loci associated with susceptibility to malaria on not only a global scale, but also between African populations within the same country borders^19^. This small-scale diversity further suggests our limited genetic data from across Africa’s ethic groups means we have only explored a cross-section of the impact malaria has had on human genetics. Despite the importance of whole genome sequencing (WGS) and GWAS studies, they remain expensive and time consuming, as well as pose a variety of ethical dilemmas when applied to vulnerable populations^20^. Recent advancements in targeted low-cost sequencing-based approaches have made it possible to target specific molecular markers on genes of interest, making use of data and knowledge that has been obtained from extensive whole genome studies^21^. This technology has already been utilised in recent studies as a method of high throughput screening for drug resistance associated loci in malaria parasites, as well as molecular markers for insecticide resistance in mosquito vectors, suggesting the capacity for a cross-species method for surveillance^21,22^.

High throughput genotyping of human genetic variants identified to have associations with severe malaria, a biological role in the process of malaria infection, or an impact on the effectiveness of treatment regimens, could be utilised to genetically profile communities with the aim of developing tailored malaria control programmes. Additionally, if combined with advancements in technologies furthering “on-the-go” science, such as Oxford Nanopore Technology’s portable MinION, these profiles could be generated in real-time and on a scale never seen before^23,24^. Here we demonstrate the versatility of custom dual-indexing technology and Illumina next generation sequencing by presenting a proof-of-concept method for profiling human genetic determinants of malarial disease in at-risk populations, utilising clinical samples from a well characterised population in Northeast Tanzania^18,25,26^.

## Results

**Figure 1:**
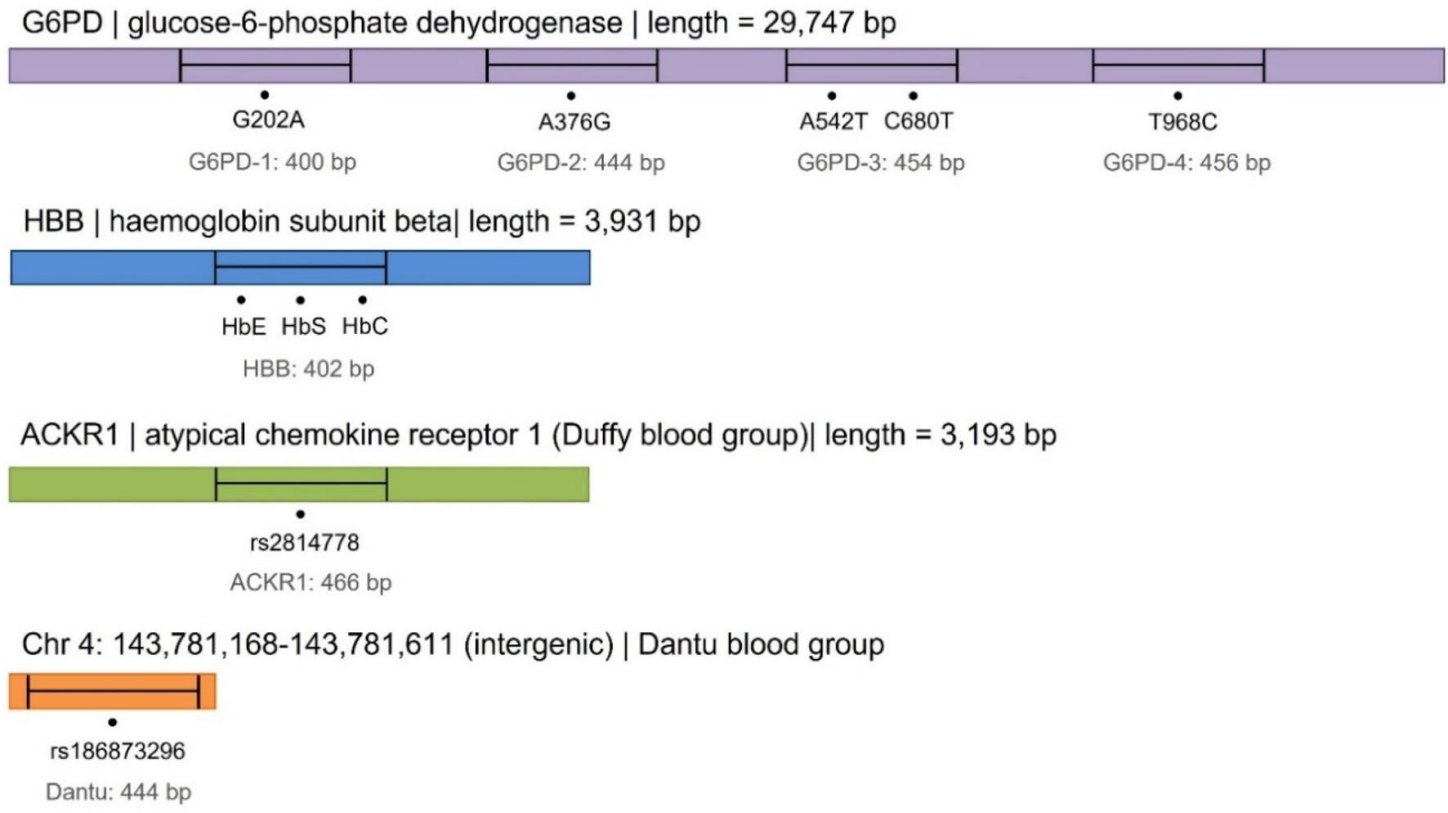
Amplicon targets for (**A**) *atypical chemokine receptor 1* (*ACKR1*; n = 1), (**B**) the intergenic Dantu genetic blood variant SNP (n = 1), (**C**) *haemoglobin subunit beta* (*HBB*; n = 1), and (**D**) *glucose-6-phosphate dehydrogenase* (*G6PD*; n = 4). Four fragments for *G6PD* were designed to encompass five SNPs of clinical relevance, while one fragment designed for each of the three remaining regions of interest.

DNA from a total of 100 patients diagnosed with severe malaria, aged between 2 months and 13 years, at the Tuele Hospital in Muheza, Tanzania were sequenced for 7 amplicon targets across 4 genes associated with malaria sensitivity and disease outcome. The genes are *HBB* (chr. 11; 1 amplicon), *G6PD* (chr. X; 4 amplicons), *atypical chemokine receptor 1* (*ACKR1/DARC*; Duffy Blood group; chr. 1; 1 ampicon), and the Dantu genetic blood variant (chr. 4; 1 amplicon) (Fig. 1). Samples were multiplexed according to sample-specific indices to promote high throughput and efficient sequencing of large sample sets. Of the 100 DNA samples that were selected for genotyping, 85 were successfully sequenced for all 7 amplicon targets. Of the 15 samples that were not completely profiled, 1 contained inadequate sequencing data for a single amplicon, 12 had inadequate sequencing data for two amplicons, and 1 had inadequate sequencing reads for all targets.

**Figure 2:**
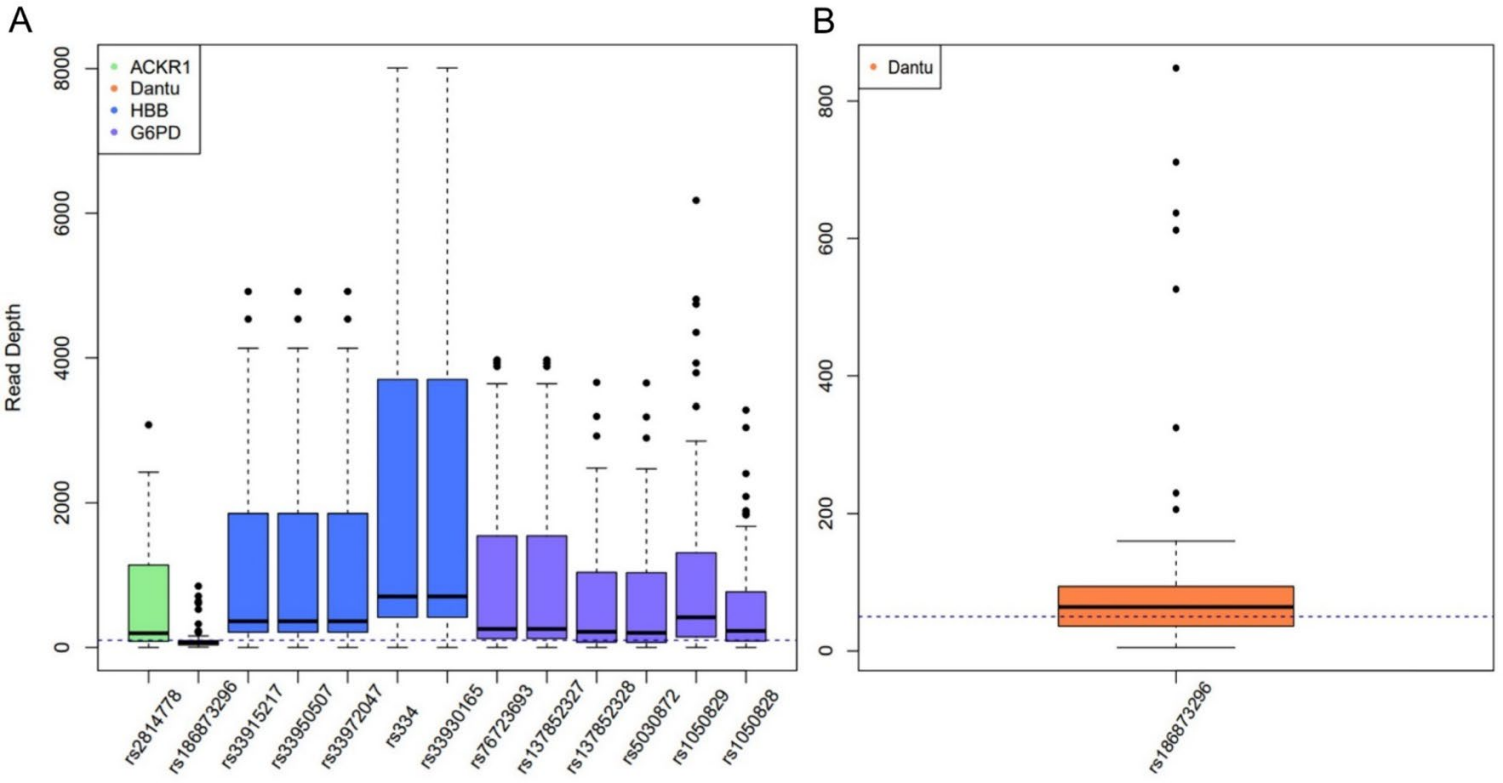
Read depth of SNPs with known associations to malaria disease severity. (**A**) Read depth of relevant SNPs on *ACKR1*, Dantu, *HBB*, and *G6PD*; trendline at 100. (**B**) Read depth of rs186873296, the intergenic Dantu genetic blood variant; trendline at 50.

The average coverage across the 7 amplicons targeting gene coding regions ranged from 973- to 2195-fold, while the coverage for the Dantu amplicon, located in an intergenic region, was markedly lower at 184-fold but was still above the recommended minimum coverage of 30-fold for human genetic analyses (Supplementary Table 1). The average read depth for specific SNPs of interest across all amplicon targets ranged from 132- to 2003-fold (Fig. 2A). The coverage for the Dantu blood group variant, rs186873296, was markedly lower than the other SNPs of interest (Fig. 2B). The lower coverage of the Dantu amplicon is likely explained by a lower primer binding affinity than the other primers used with this method^27^.

### Variants characterised on HBB, ACKR1, and the Dantu blood variant

A single amplicon was used to characterise variants on the *HBB* gene. The rs334 variant, associated with sickle cell trait (HbS), and the rs33950507 (HbE) and rs33930165 (HbC) variants were used to assess the accuracy of amplicon sequencing and variant calling for *HBB* as pre-existing genotyping data was available. The rs334 variant was recorded in 9.1% (9/99) of participants with 5 individuals identified as heterozygous for the sickle cell allele, carrying both the variant allele and wild-type allele (HbAS), and 4 individuals identified as homozygous positive for the sickle cell allele (HbSS; sickle-cell disease). As expected, the rs33950507 variant (HbE) was not identified in any of the participants in this study, as this is specific to Southeast Asian populations. Similarly, the rs33930165 variant (HbC) was not identified in any participants, consistent with its high abundance in areas of West Africa. Pre-existing genotyping data was available for rs334, rs33950507 and rs33930165; amplicon sequencing data matched the pre-existing genotyping data for all screened individuals (100% concordance) (Table 1). Off-target variants were also classified and presented with their corresponding median read depth (see Table 1).

**Table 1:** Distribution of alleles associated with malaria disease severity, as well as off-target non-synonymous variants, identified on *HBB, G6PD*, Dantu, and *ACKR1* loci in Northeast Tanzania using amplicon sequencing.

Gene	Chrom	Position	rs ID^a^	Variant information	Homozygous negative (−/−)		Heterozygous (−/+)		Homozygous positive (+/+)		Mean DP	Concordance^b^ %
*G6PD*	X	154532738	rs2230036	C>T	CC	87	CT	6	TT	6	803	*NA*
		154533025	rs76723693	968 A>G	AA	99	AG	0	GG	0	798	100
		154533122	rs137852327	C>T	CC	99	CT	0	TT	0	798	*NA*
		154534125	rs137852328	680 C>A	CC	99	CA	0	AA	0	629	100
		154534177	rs5986875	G>A	GG	98	GA	1	AA	0	629	*NA*
		154534440	rs5030872	542 T>A	TT	99	TA	0	AA	0	622	100
		154535443	*NA*	G>A	GG	86	GA	1	AA	0	622	*NA*
		154535468	*NA*	G>T	GG	86	GT	1	TT	0	622	*NA*
		154534527	*NA*	T>C	TT	86	TC	1	CC	0	622	*NA*
		154535277	rs1050829	376 T>C	TT	48	TC	16	CC	23	956	96.6
		154536002	rs1050828	202 C>T	CC	71	CT	8	TT	8	530	100
*HBB*	11	5226867	*NA*	C>G	CC	98	CG	1	GG	0	1020	*NA*
		5226925	rs33915217	β + thal	CC	99	CA	0	AA	0	1020	*NA*
		5226932	rs35578002	G>T	GG	98	GT	1	TT	0	1020	*NA*
		5226943	rs33950507	HbE	CC	99	CT	0	TT	0	1020	100
		5226963	rs33972047	β + thal	TT	99	TC	0	CC	0	1020	*NA*
		5226966	rs35382661	A>C	AA	97	AC	2	CC	0	1118	*NA*
		5227002	rs334	HbS	TT	90	TA	4	AA	5	2003	100
		5227003	rs33930165	HbC	CC	99	CT	0	TT	0	2003	100
		5227013	rs713040	β + thal; HPFH^c^	AA	4	AG	27	GG	65	2002	*NA*
		5227072	rs386134236	A>G	AA	98	AG	1	GG	0	984	*NA*
*ACKR1*	1	159204646	*NA*	A>C	AA	76	AC	19	CC	0	644	*NA*
		159204893	rs2814778	Fy(a-b-) T>C	TT	0	TC	0	CC	95	623	100
*Dantu*	4	143781321	rs186873296	Intergenic A>G	AA	95	AG	3	GG	0	132	99.0
		143781342	*NA*	G>T	GG	98	GT	1	TT	0	184	*NA*

The *HBB* amplicon encompassed several other polymorphisms not categorised in pre-existing genotyping data in this study population, including rs33972047, rs33915217, and rs713040. The rs713040 variant, associated with benign presentations of beta thalassemia (β + thal) and the fetal haemoglobin quantitative trait locus 1, was identified in 95.8% (92/96) of participants, with 67.7% (65/96) of individuals being homozygous positive for the variant allele. The rs33972047 and rs33915217 variants were not identified in any participants.

The variant rs2814778 on *ACKR1*, which encodes the Duffy blood group antigen, results in a Duffy-negative phenotype, which is predominantly fixed in most populations across Sub-Saharan Africa (Table 2). The average coverage for the *ACKR1* amplicon was 1226-fold and, as expected the rs2814778 variant was identified in 100% (95/95) of study participants and matched pre-existing genotyping data for all individuals. The Dantu blood group variant rs186873296 was identified in 3.1% of study participants (3/98) with three individuals being heterozygous for the variant allele. There was one individual (1/98) that was misidentified as homozygous negative for the variant allele using amplicon sequencing that had been previously identified as heterozygous for the variant allele in the pre-existing genotyping data.

**Table 2:** Allele frequencies of variants associated with malaria disease severity on *HBB, G6PD*, Dantu, and *ACKR1* loci in Northeast Tanzanian, African, European, and global human populations.

Gene	rsID	Amplicon Seq^a,b^		Controls^a^ (*n* = 477)	Cases^a,b^ (*n* = 506)	African		European		Global	
		%	*n*			%	*n*	%	*n*	%	*n*
*G6PD*	rs1050828	18.4	87	20.0	16.3	13.5	1003	0	766	3.8	3775
	rs1050829	44.8	87	38.5	37.4	33.8	1003	0.4	766	9.5	3775
	rs5030872	0	99	0	0	1.1	3712	0	69,444	< 0.1	79,538
	rs137852328	0	99	0	0	0	2714	< 0.01	13,108	< 0.01	17,548
	rs76723693	0	99	0	0	1.0	1003	0	766	3.2	3775
	rs5986875	1.0	99	*NA*	*NA*	3.6	1003	0	766	1.0	3775
	rs137852327	0	99	*NA*	*NA*	0	1003	0	766	0.2	3775
	rs2230036	12.1	99	*NA*	*NA*	12.2	1003	0	766	3.3	3775
*HBB*	rs334	9.1	99	16.5	2.0	10.0	1322	0	1006	2.7	5008
	rs33930165	0	99	0	0	1.3	1322	0	1006	0.3	5008
	rs33950507	0	99	0	0	0	1322	0	1006	0.3	5008
	rs33972047	0	99	*NA*	*NA*	0	1690	0	13,150	0	15,924
	rs33915217	0	99	*NA*	*NA*	0	1322	0	1006	0.1	5008
	rs713040	95.8	96	*NA*	*NA*	88.4	1322	83.0	1006	71.4	5008
*ACKR1*	rs2814778	100	95	*NA*	*NA*	96.4	1322	0.6	1006	26.6	5008
Dantu	rs186873296	3.1	98	*NA*	*NA*	0.4	1322	0.1	1006	0.1	5008

^a^Muheza, Tanzania.

^b^Clinically severe malaria (described in “Materials and methods”).

### Genotyping for G6PD deficiency

**Figure 3:**
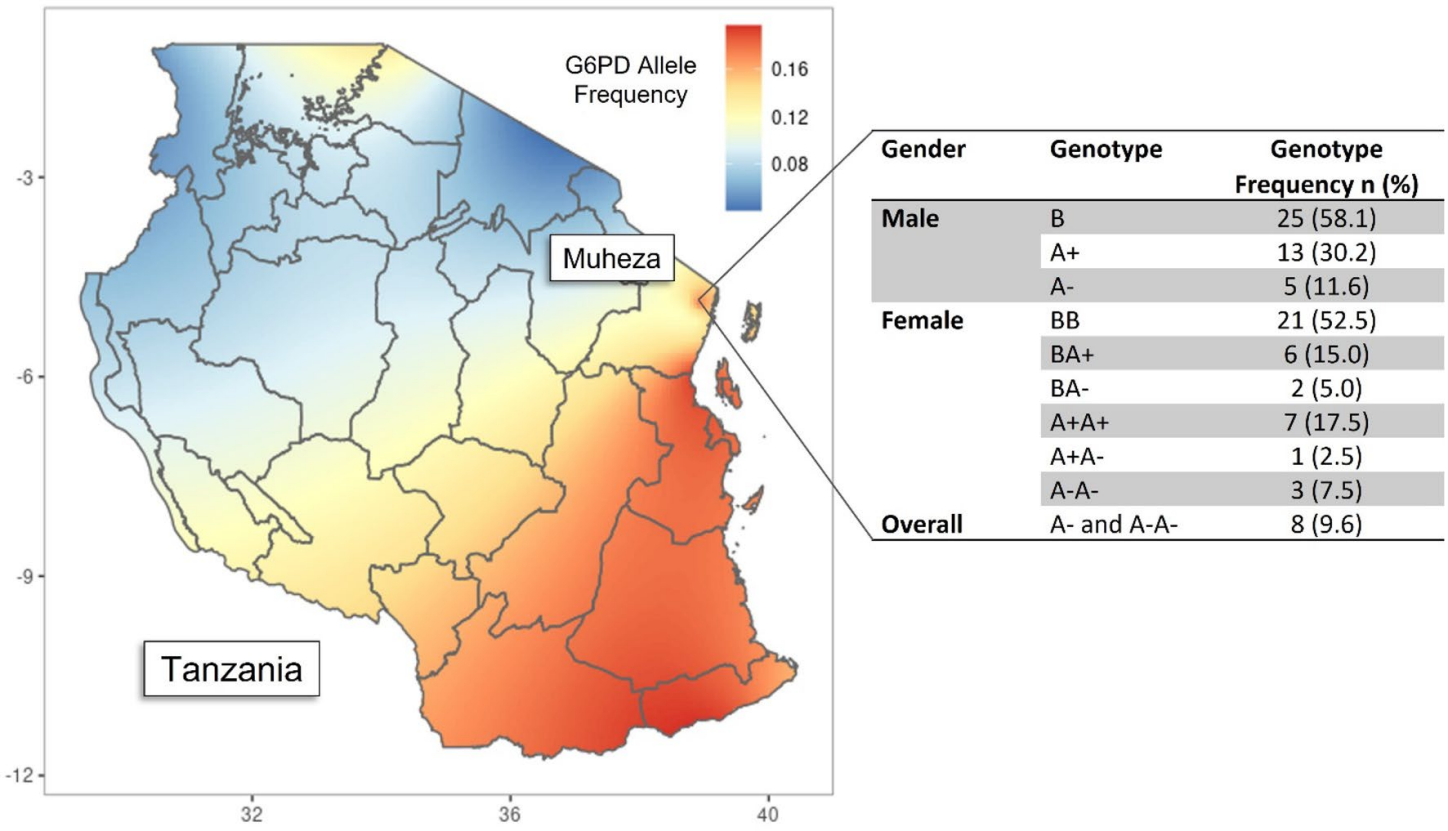
Distribution of G6PD allele frequency and genotypes across Tanzania. (**A**) G6PD variant allele frequency distribution across Tanzania^39^. (**B**) Frequency of G6PD genotypes among male and female children with severe malaria from Tuele Hospital (Muheza, Tanzania) determined using amplicon sequencing as a method of genotyping. G6PD Genotype: male normal = A+ or B; male hemizygous = A−; female normal = BB or BA+ or A+A+; female heterozygous = BA− or A+A−; female homozygous = A−A−. Severe G6PD deficiency genotypes = A− and A−A−.

*G6PD* was covered by four amplicons and overall had high sequencing coverage: average coverage of 1089-, 973-, 1212-, and 1548-fold, respectively. The resulting amplicon sequencing data was used to determine the G6PD genotype of participants (Table 1) and infer the G6PD A-deficiency phenotype of individuals based on alleles present at nucleotide positions 202 (rs1050828) and 376 (rs1050829), as well as others (542, rs5030872; 680, rs137852328; 968, rs76723693) (Fig. 3). The rs1050828 variant was identified in 18.4% (16/87) of participants, with 8 individuals identified as homozygous positive for the variant allele and 8 individuals identified as heterozygous carriers, while the rs1050829 variant was identified in 44.8% (39/87) of participants, with 26.4% (23/87) being homozygous positive for the variant allele.

Amplicon sequencing genotyping data for rs1050828 matched pre-existing genotyping data for all screened individuals (87/87). There were 2 individuals that were misidentified as heterozygous for the rs1050829 variant allele that had previously been identified as homozygous negative, as well as one individual that was misidentified as heterozygous the rs1050829 allele that had previously been identified to be homozygous positive. The 542, 680, and 968 variants were not identified, however two additional variants, both believed to be benign variants of G6PD deficiency, were identified within the sample population, rs2230036 (12.1%; 12/99) and rs5986875 (1%; 1/99).

Of the 83 individuals with accurate amplicon sequencing genotyping data, available for both the 202 and 376 nucleotide positions, most participants (55.4%; 46/83) were identified to have the wild-type G6PD genotype (Males = B hemizygous; Females = BB homozygous) (Table 3). There were 8 individuals (9.6%), 5 males and 3 females, identified to have the severely deficient G6PD phenotype (Males = A−; Females = A−A−).

**Table 3:** Frequency of G6PD genotypes* among male and female children with severe malaria from Tuele Hospital (Muheza, Tanzania) determined using amplicon sequencing as a method of genotyping.

Gender	Genotype	Frequency N (%)	
Male	B	25	58.1
	A+	13	30.2
	A−	5	11.6
Female	BB	21	52.5
	BA+	6	15.0
	BA−	2	5.0
	A+A+	7	17.5
	A+A−	1	2.5
	A−A−	3	7.5
Overall	A− and A−A−	8	9.6

*G6PD Genotype: male normal = A+ or B; male hemizygous = A−; female normal = BB or BA+ or A+A+; female heterozygous = BA- or A+A−; female homozygous = A−A−. Severe G6PD deficiency genotypes = A− and A−A.

## Discussion

With the stalling progress of malaria control programmes aiming for global elimination, molecular surveillance tools offer a next-generation solution to a major public health burden^1^. Despite the fact that malaria has exhibited the strongest known selective pressure on the human genome in recent history, and its disproportionate public health impact in sub-Saharan Africa, most of the African continents highly diverse ethnic groups are underrepresented in genetic studies^13^. Not only does this limit our understanding of the evolution and distribution of human genetic variants associated with malaria disease severity across the continent, but it also increases the difficulty of successfully delivering appropriately tailored public health or personalised medicine interventions. One of the main barriers to expanding human genetic profiling to sub-Saharan Africa, behind cost and infrastructure, has been the ethical implications of carrying out WGS and GWAS on vulnerable populations and the difficulties associated with safely storing vast quantities of personal and genomic data for those individuals^15,20^.

Advancements in low-cost targeted sequencing technology have made it possible to cheaply target specific genes of interest, eliminating the need to perform WGS or store extraneous human genomic data. Here we presented a proof-of-concept method for the targeted sequencing of human genetic variants associated with malaria disease severity and treatment efficacy using an Illumina sequencing platform and custom dual-indexing technology. The custom 5′- and 3′-indices allowed for individual sample identification across multiple targets following pooling and sequencing in a single reaction. For this study, 10 unique forward indices and 10 unique reverse indices were designed with 8 base pairs each, creating 90 possible dual index combinations. This number is infinitely expandable depending on the requirements of the study. This method was used to sequence and screen for genetic variants on *G6PD, HBB*, and *ACKR1* genes, as well as the Dantu genetic blood variant. In this study, severe malaria cases were chosen with an enrichment for the sickle-cell allele, to assist with validation of amplicon sequencing accuracy, therefore the HbS allele frequency in our dataset (9.1%; 9/99) does not reflect the anticipated population frequency (16.5%; 79/477). For other variants classified within this study, the allele frequencies are broadly similar between the study population and estimates from both Tanzanian and other sub-Saharan African populations.

Due to the size of the *G6PD* gene, located on the X chromosome, four amplicons were used to target five key variants (202, 376, 542, 680, and 968) associated with G6PD deficiency, namely African-type G6PD A−, which results in diminished activity of the enzyme glucose-6-phosphatase dehydrogenase enzyme^11,28^. All four amplicons were observed to have high sequencing coverage (average > 950-fold). The 202 and 376 G6PD variants are common in sub-Saharan Africa, whereas 542 and 968 variants have been observed in West African populations (e.g., The Gambia) and the 680 variant appears rare in the continent^7,29^. This trend was observed in our data where the 202 and 376 variant alleles were found in 18.4% and 44.8% of study participants, respectively, while the 542, 680, and 968 variants were not identified. Through performing high-resolution sequencing of genetic targets, rather than genotyping for specific loci only, this methodology has the capacity to capture novel, or rare, polymorphisms, such as those with functional consequences that would normally be missed, as well as potentially explain allelic heterogeneity, often confounded in genetic association studies^29^. Two additional variants (rs2230036, rs5986875), both believed to be benign variants of G6PD deficiency, were identified alongside the previously described variants.

Using this method, two individuals were misidentified as being heterozygous for the 376-variant allele, as opposed to being homozygous negative for the allele, and one person was misidentified as heterozygous positive for the allele rather than homozygous positive. These misidentifications could be due to cross-contamination with other samples during the high throughput PCR step of this process. In most instances, small amounts of contamination with low read counts will be removed by bioinformatic filtering steps during downstream analysis, alongside other sequencing artifacts**.** Overall, discordance between amplicon sequencing data and available genotyping data was extremely low. Across our study participants, high quality sequencing data was achieved for 962 genotypes with pre-existing data. Of these 962 genotypes, only 4 were discordant (discordance: 0.42%, 4.2 per 1000 genotypes), including the 3 positions discussed on G6PD. We present this discordance to highlight the need for further testing and optimisation in larger datasets, across multiple populations, before this methodology could be applied within a clinical setting.

For the *HBB* gene, one amplicon was designed to target variants associated with thalassaemias, sickle cell anaemia (HbS), HbC, HbE, as well as a variety of other haemoglobinopathies, and the average coverage for the HBB amplicon was 2195-fold. The HbS allele was identified in 9.1% of study participants with 5 individuals identified to have both the variant allele and wild-type allele (HbAS) and 4 individuals identified to have both variant alleles (HbSS) which results in the clinical manifestation of sickle cell disease rather than conferring a relatively harmless protective effect^30,31^. The HbC and HbE alleles were not observed in this study population which was to be expected as the HbC allele is more commonly found in people of West African descent and the HbE allele is common in Southeast Asian populations^30,32^. The rs713040 variant, associated with benign presentations of beta thalassemia and the hereditary persistence of foetal haemoglobin, was identified in 95.8% of participants, however there is no known impact of this variant associated with malaria infection or disease.

A single amplicon was used to sequence the rs2814778 variant on the *ACKR1* gene, which encodes the Duffy blood group antigen located on the surface of red blood cells^33^. The rs2814778 variant results in a Duffy negative phenotype, or the absence of the Duffy antigen, and is generally fixed in sub-Saharan African populations due to its ability to protect against *P. vivax* infection, which relies on the protein as a receptor for invasion into the red blood cells, and the lack of any known pathogenic effects in humans who harbour this variant^9,33^. As anticipated, 100% of study participants were identified to be homozygous positive for the rs2814778 variant, which results in the Duffy negative phenotype^9^.

The amplicon designed to target the Dantu blood group variant was the only amplicon designed to target an intergenic region. The Dantu blood variant is located on chromosome 4, upstream of the *GYPA* and *GYPB* genes, and was recently identified as the causative polymorphism behind a novel protein expressed on the surface of red blood cells that alters the red blood cell surface tension and makes it more difficult for malaria parasites to invade^34^. To date there is no known evidence of health complications in carriers of this variant allele^34^. The Dantu variant was identified in 3.1% of study participants, with 3 individuals being heterozygous for the variant allele. There was one individual that was misidentified as homozygous negative for the variant allele using amplicon sequencing that had been previously identified as heterozygous for the variant allele in the pre-existing genotyping data. This misclassification was due to low sequencing reads of the non-reference allele, resulting in the alternate allele not meeting the filtering threshold requiring > 20% of reads to be non-reference for a heterozygous classification^35^.

The average coverage for the Dantu amplicon was 184-fold which was substantially lower than the other amplicons used in this method. This lower coverage is likely due to lower primer binding affinity when compared to other primers used in this method. All primer sets used in this study were designed to have the same annealing temperatures so all reactions could be run concurrently using one PCR programme. This reduces the overall time and costs associated with processing high volume datasets. A lower annealing temperature may increase the overall coverage of the Dantu amplicon, however the coverage achieved using the annealing temperature described in our methodology achieved sufficient coverage to perform confident variant calling^36^.

The successful genotyping of targets across *G6PD, HBB, ACKR1,* and the Dantu blood variant, highlights the versatility of amplicon sequencing and suggests a capacity for easy expansion to other genetic markers of disease. Such targets could include the *ABO* gene, variants linked to drug metabolism, or polymorphisms yet to be identified by future GWAS studies. A cost-effective and highly adaptable genotyping assay, such as the one presented here, has the potential to assist with surveillance and personalised medicine, while simultaneously addressing important ethical concerns surrounding the collection of human data and the need for low-cost sequencing methods to be accessible for low- and middle-income countries to promote in-country research capacity.

## Conclusion

This study presents a methodology that makes use of advancements in high throughput sequencing, and custom-dual indexing technology, to genetically profile human genetic variants. We focused on variants associated with malaria disease severity and treatment implications in genetically diverse communities, simultaneously cutting down on extraneous data collected through genome-wide studies, as well as the associated costs. This methodology leverages off previously presented techniques used in characterising drug resistance biomarkers in *P. falciparum* infections and suggests the possibly of a cross-species method of surveillance which, together, could inform region-specific, highly specialised, malaria intervention programmes.

## Materials and methods

### Study site description and sample collection

Human and parasite DNA used in this study was obtained from samples collected in a study conducted in Tanga region, Northeast Tanzania between June 2006 and May 2007 [PMID: 25671784; PMID: 29381699]. Samples had been recruited from individuals, aged between 2 months and 13 years, admitted to Tuele Hospital in Muheza, with severe malaria. Severe malaria cases were defined as those with a history of fever within the 48 h prior to admission, asexual *P. falciparum* parasitaemia, and one or all of the following: more than 2 seizures within the previous 24 h; Blantyre coma score (BCS) of less than 3 (repeated if BCS < 5 and convulsion within 1 h of anticonvulsant given within 6 h); prostration (the inability to sit unsupported or, if aged < 8 months, to drink); respiratory distress (deep breathing, low chest wall indrawing, a respiratory rate greater than 70 bpm, or an oxygen saturation of less than 90%); jaundice (identified by inspection of sclera); severe anaemia (haemoglobin less than 5 g/dl), blood glucose level less than 2.5 mmol/l, or a blood lactate greater than 5 mmol/l.

Individuals included within the study underwent genome-wide and targeted genotyping^7,18,37^. Genotyping was carried out to identify variants with previously reported associations to malaria severity, as well as a biological role in malaria infection and disease, was carried out for *G6PD*, *HBB*, *ACKR1*, and the Dantu blood variant (Table 1). Genotyping data specifically relevant to this study was available, including for *G6PD* (e.g., rs1050828, rs1050829, rs5030872, rs137852328, rs76723693), *HBB* (e.g., rs334, rs33930165, rs33950507), *ACKR1* (e.g., rs2814778), and the Dantu blood group variant (rs186873296)^18,25^. This data was used to assess the sequencing and variant calling accuracy of this methodology. All experimental protocols were approved by the ethical review board of the London School of Hygiene and Tropical Medicine and the Tanzanian National Medical Research Institute (Proposal number: ID 4093). All methods were carried out in accordance with relevant guidelines and regulations and informed consent was obtained from all subjects and/or their legal guardian(s).

### Study population characteristics

The population of Muheza is dominated by the Mzigua and Wasambaa ethnic groups which generally rely on subsistence agriculture, livestock keeping, and fishing as their main sources of income. At the time of sample collection, much of the population had access to primary health facilities, however medicine shortages, high costs for users, and inadequate facility infrastructure limited the scope of these facilities. At the time of collection, transmission of *Plasmodium falciparum* was recorded to be high (~ 50–700 infected bites per person per year) and followed a seasonal pattern with two distinct seasonal peaks per year. In 2002, community prevalence of *P. falciparum* was 88.2% in children aged 2 to 5 years of age. The dominant malaria transmitting vectors within the area were recorded to be *Anopheles gambiae *sensu stricto and *Anopheles funestus*^38^.

### Primer design

Primers used in this study were designed to investigate polymorphisms on human genes known to be associated with malaria disease severity and outcomes of disease by amplifying fragments, or amplicons, of between 400 and 600 base pairs (bp). Targeted genes included: (i) *HBB* (rs334 (HbS), rs33950507 (HbE), rs33930165 (HbC), rs33941377, rs33944208, rs33972047); (ii) *ACKR1* (rs2814778); (iii) *G6PD* (rs1050828, rs1050829, rs78365220, rs5030872, rs137852328, rs137852314, rs5030868, rs137852330, rs76723693, rs13785232), (iv) Dantu blood group (rs186873296). Forward and reverse primers included unique 8 bp indices, or barcodes, used to demultiplex sequencing data, described below. The unique primer indices were designed to be 8 bp, to mitigate chances of recombination and contamination possible with shorter indices. Sequencing adaptors were added at Illumina using adaptor ligation technology.

### PCR reactions

PCRs were performed using a master mix containing 5 µl of Q5 Reaction Buffer (New England BioLabs), 0.5 µl of dNTPs (1n nM stocks, New England BioLabs), 0.25 µl Q5 Hot Start High-Fidelity DNA Polymerase (New England BioLabs), and 15.75 µl Milli-Q water (Merck). For each reaction, a total of 1.25 µl of forward and 1.25 µl of reverse primer (10 pmol/µl stocks) were used with 1 µl of DNA for a total reaction volume of 25 µl. The reactions were carried out in a thermocycler consisting of the following steps: Heat activation for 15 min at 72 °C, 30 cycles of denaturation for 20 s at 95 °C, annealing for 2 min at 60 °C, elongation for 2 min at 72 °C, and a final elongation for 10 min at 72 °C, followed by a hold at 10 °C.

### Amplicon purification and pooling

Combinations of indices were conserved by sample identifier, regardless of amplicon target. Samples without overlapping combinations of indices were pooled together for cleaning and sequencing, with a maximum of 200 amplicons allowed per pool to ensure sufficient sequencing coverage. Pooled samples were cleaned prior to sequencing using KAPA bead purification, following the manufacturer’s instructions. A ratio of 1:0.70 of product to bead volume was used to size select DNA fragments the size of amplicons and remove excess primers. DNA was measured (Qubit dsDNA HS) and normalised to 20 ng per 25 µl.

### Illumina sequencing and bioinformatics

One hundred human DNA samples were chosen from severe malaria cases (Muheza, Tanzania) and sequenced following PCR amplification of all the amplicon targets. Sequencing was carried out with the Illumina MiSeq platform using adaptor ligation technology at Genewiz (GENEWIZ Germany GmbH). Following demultiplexing of pooled sequencing reads according to the unique sample IDs, the raw sequencing data was mapped to the GRCh38.p13 *Homo sapiens* reference genome using the default parameters set for Illumina data within the software *bwa-mem*. SNPs and indels were called using the *samtools*, freebayes, and GATK software suites. Samples with a sequencing coverage < 30-fold for positions of interest were discarded and not used in further data interpretation^36^. Samples with < 20% of reads for a non-reference allele were classed as homozygous-negative and sample with > 80% of reads for a non-reference allele were classed as homozygous-positive. SNPs were annotated using the *SnpSift* software, and information available via ClinVar, to identify variants with known rsID numbers (reference SNP cluster ID).

### Genotyping data

The sequence data were compared to genotyping data on the samples from previous work, allowing an assessment of error rates. This includes candidate SNPs for *G6PD* (202 rs1050828, 376 rs1050829, 542 rs5030872, 680 rs137852328, 968 rs76723693), *ACKR1* (Duffy blood group antigen, including rs2814778) and *HBB* (HbS rs334, HbC rs33930165, and HbE rs33950507)^7,25,37^, as well as imputed genome-wide polymorphism (from Illumina Omni 2.5 million SNP chip)^18^.

## Supplementary Information

Supplementary Information.

## Data Availability

All raw sequence data is available from the ENA (project accession number PRJEB58734).
