# Supplementary material for: High throughput human genotyping for variants associated with malarial disease outcomes using custom targeted amplicon sequencing
Source: Sci Rep. 2023 Jul 26;13:12062. doi: 10.1038/s41598-023-39233-z (PMC10371994; doi:10.1038/s41598-023-39233-z)

SUPPLEMENTARY INFORMATION

Table S1: Amplicon coverage across 98 host DNA samples. Average, minimum, and maximum read count per target amplicon.

Amplicon	Average (*n* = 99)	Minimum	Maximum
G6PD-1	1089	21	3857
G6PD-2	973	9	3572
G6PD-3	1212	50	3890
G6PD-4	1548	77	4216
HBB	2195	112	5917
ACKR1	1226	97	5292
Dantu	184	5	2729

Table S2: Primers

Gene	RS SNP ID	Primer Sequence
ACKR1	rs2814778	Forward: TGTGCTTGAAGAATCTCTCCTT<br>Reverse: CAGGGGAAATGAGGGGCATAG
G6PD-1	rs1050828	Forward: CTGGTAGAGAGGGCAGAACC<br>Reverse: GACATGCTTGTGGCCCAGTA
G6PD-2	rs1050829, rs78365220	Forward: CGCTCATAGAGTGGTGGGAG<br>Reverse: CACTGACTTCTGAGGGCACC
G6PD-3	rs5030872, rs137852328, rs137852314, rs5030868, rs137852330	Forward: GGATAACGCAGGCGATGTTG<br>Reverse: TGATCCTCACTCCCCGAAGA
G6PD-4	rs76723693, rs137852327	Forward: GCCGGCCACATCATGGAAC<br>Reverse: CAACTCAACACCCAAGGAGCC
HBB	rs334, rs33950507, rs33972047, rs33930165, rs33941377, rs33944208	Forward: TGGGAAAATAGACCAATAGGCAGA<br>Reverse: AAGGACAGGTACGGCTGTCA
*Dantu**	rs186873296	Forward: GCAGATTAGCATTCACCCAG<br>Reverse: TGCTCCAGAGTAAGCATCCTTC

*Intergenic region

Figure S1: Flow chart describing the bioinformatic pipeline of analysis from raw sequencing reads to variant calling.

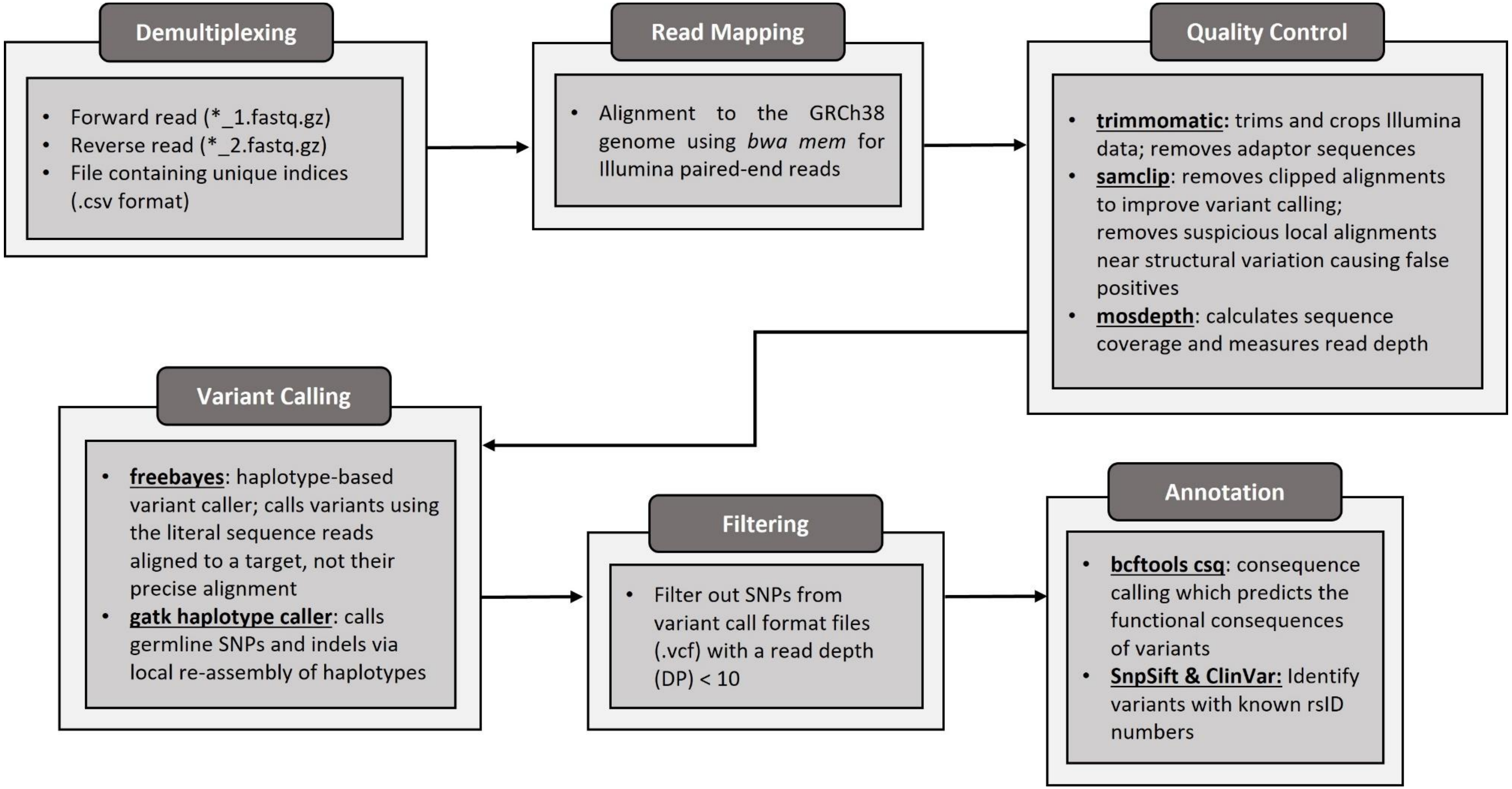

Supplement: Supplementary file 1 — Supplementary Information. [file 41598_2023_39233_MOESM1_ESM.pdf]
